# Integrating multi-omics and machine learning strategies to explore the “gene-protein-metabolite” network in ischemic heart failure with Qi deficiency and blood stasis syndrome

**DOI:** 10.1186/s13020-025-01151-9

**Published:** 2025-07-17

**Authors:** Jingjing Wei, Aolong Wang, Peng Yu, Yang Sun, Wenjun Wu, Yilin Zhang, Rui Yu, Bin Li, Mingjun Zhu

**Affiliations:** 1 Department of Cardiovascular Disease, The First Affiliated Hospital of Henan University of Chinese Medicine, Renmin Road 19, Jinshui District, Zhengzhou, 450000 China; 2 Department of Orthopedics, The First Affiliated Hospital of Henan University of Chinese Medicine, Zhengzhou, 450000 Henan China

**Keywords:** Ischemic heart failure, Yiqi Huoxue, Multi-omics, Machine learning, Molecular docking, Traditional Chinese medicine

## Abstract

**Background:**

Ischemic heart failure (IHF) is a multifaceted syndrome associated with significant mortality and high hospitalization rates globally. According to traditional Chinese medicine (TCM) theory, Qi Deficiency and Blood Stasis (QXXY) Syndrome serves as the pathological basis of IHF. This study aims to investigate the biological basis of QXXY syndrome in IHF patients through an integrated multi-omics approach.

**Methods:**

We enrolled 100 participants, comprising 40 IHF patients with QXXY syndrome (IHF-QXXY), 40 IHF patients without QXXY syndrome, and 20 healthy controls. Utilizing an integrated approach combining RNA sequencing (RNA-seq), data-independent acquisition (DIA) proteomics, and targeted metabolomics, we established a comprehensive “gene-protein-metabolite” network for IHF-QXXY syndrome. Candidate biomarkers were identified through machine learning algorithms and further validated using RT-qPCR and targeted proteomics via intelligent parallel reaction monitoring (iPRM).

**Results:**

Patients with IHF-QXXY syndrome present with pronounced disruptions in energy metabolism, chronic inflammation, and coagulation abnormalities. The “gene-protein-metabolite” network of IHF-QXXY syndrome comprises six mRNAs, four proteins, and five metabolites. Key pathways involve the activation of neutrophil extracellular traps formation, platelet activation, the HIF-1 signaling pathway, and glycolysis/gluconeogenesis, alongside the suppression of the citrate cycle and oxidative phosphorylation. The key metabolites potentially associated with QXXY syndrome include 3-methylpentanoic acid, arachidonic acid, N-acetylaspartylglutamic acid, L-acetylcarnitine, and 12-hydroxystearic acid. We identified a panel of candidate biomarkers, including HIF-1α, IL10, PAD4, ACTG1, SOD2, GAPDH, FGA, FN1, F13A1, and ATP5PF. This biomarker combination significantly enhanced the diagnostic performance of IHF-QXXY syndrome (AUC > 0.863) and retained high diagnostic accuracy during validation (AUC > 0.75).

**Conclusion:**

This study provides a comprehensive characterization of the molecular features of QXXY syndrome in IHF patients, highlighting key pathways and biomarkers linked to energy metabolism dysregulation, chronic inflammation, and coagulation abnormalities. These findings may provide novel insights and methods for further advancing this research field.

**Graphical Abstract:**

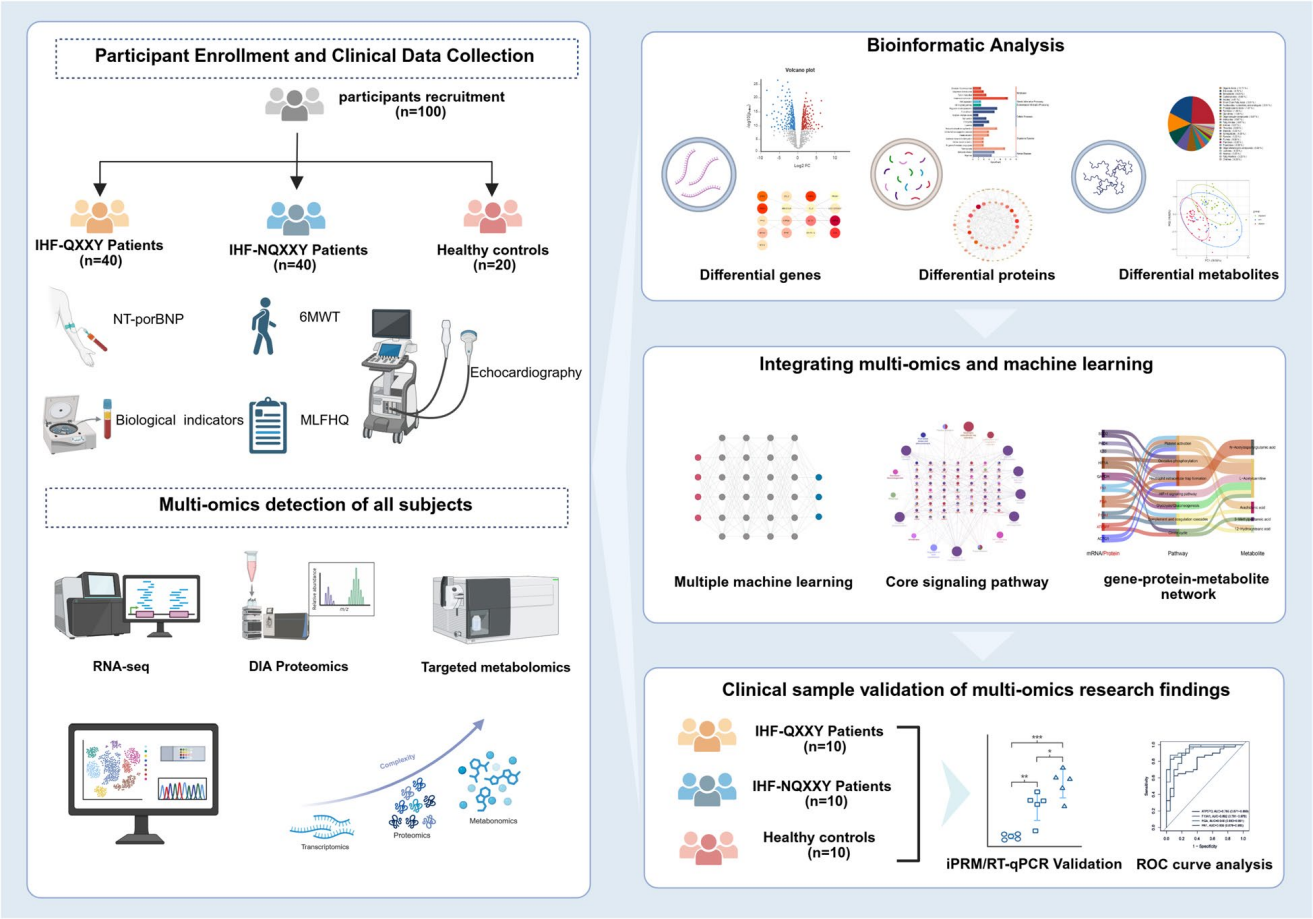

**Supplementary Information:**

The online version contains supplementary material available at 10.1186/s13020-025-01151-9.

## Introduction

Heart failure (HF) remains a leading global cause of morbidity and mortality, with its burden escalating due to an aging population. A 2021 large-scale epidemiological study revealed that the standardized prevalence of HF among individuals aged 25 and older in China is 1.10%, translating to an estimated 12.1 million cases and nearly 3 million new cases annually [1]. The average annual hospitalization rate for HF patients is 3.3 times per individual, with an associated cost of $4,406.8 per person per year. In the United States, over 6 million individuals are affected by HF, and its prevalence is projected to increase by 46% by 2030, impacting approximately 8 million adults [2]. Ischemic heart disease (IHD) is a predominant contributor to HF, accounting for nearly 70% of cases [3]. IHD elevates the risk of HF by eightfold, and despite concerted efforts to mitigate key risk factors, the prognosis for patients with ischemic heart failure (IHF) remains dismal. This underscores the critical need for a deeper understanding of the underlying pathophysiology and the development of innovative diagnostic and therapeutic strategies.

Traditional Chinese Medicine (TCM) has been widely utilized in the management of IHF, with the concept of “Qi deficiency and blood stasis syndrome” (QXXY syndrome) being particularly relevant [4–6]. QXXY syndrome is considered the primary TCM syndrome type of IHF, characterized by clinical manifestations such as fatigue, shortness of breath, and palpitations. Theoretical perspectives suggest that these symptoms result from impaired energy metabolism, microcirculatory dysfunction, and chronic inflammation. However, the biological underpinnings of QXXY syndrome in IHF remain poorly elucidated, hindering its integration into contemporary medical practice.

Recent advancements in multi-omics technologies, including transcriptomics, proteomics, and metabolomics, have revolutionized the exploration of molecular mechanisms underlying complex diseases. By integrating these approaches, it is possible to construct comprehensive “gene-protein-metabolite” networks that capture the intricate interactions driving disease progression [7]. These networks can unveil key biomarkers and pathways associated with specific disease phenotypes, offering novel insights into disease mechanisms and potential therapeutic targets. In recent years, the integration of multi-omics methodologies and computational biology has garnered significant attention for elucidating the mechanisms of TCM syndromes. For instance, Jie Wang et al. employed an integrated strategy combining RNA-seq, data-independent acquisition (DIA) proteomics, and untargeted metabolomics to analyze 90 clinical samples of coronary heart disease, constructing a “gene-protein-metabolite” network for coronary heart disease with phlegm and blood stasis syndrome [8]. Similarly, Weidong Zhang et al. utilized a combined approach of proteomics, metabolomics, and network pharmacology to systematically investigate the biological basis of two TCM syndromes in coronary heart disease: Cold Congealing and Qi Stagnation (CCQS) and Qi Stagnation and Blood Stasis (QSBS) [9]. Despite these advancements, significant challenges persist in understanding QXXY syndrome in IHF, including reliance on single-omics approaches, limited depth of studies, inability to cross-validate findings, and insufficient validation of conclusions. Collectively, a multi-omics approach holds immense potential to elucidate the molecular signatures of QXXY syndrome, thereby bridging the gap between TCM theory and modern biomedical science.

In this study, we employed an integrated multi-omics strategy combined with computational biology to investigate the biological basis of QXXY syndrome in IHF patients. We enrolled 100 participants, comprising 40 IHF patients with QXXY syndrome (IHF-QXXY), 40 IHF patients without QXXY syndrome (IHF-NQXXY), and 20 healthy controls (HC). Utilizing high-throughput transcriptomic sequencing, DIA proteomics, and targeted metabolomics, alongside advanced machine learning techniques, we identified differentially expressed genes (DEGs), proteins (DEPs), and metabolites (DMs) associated with QXXY syndrome. These findings were further validated using RT-qPCR and intelligent parallel reaction monitoring (iPRM) targeted proteomics. Our primary objective was to construct a “gene-protein-metabolite” network that delineates the unique molecular landscape of IHF-QXXY, thereby laying the groundwork for the development of diagnostic biomarkers and targeted therapies.

This study represents the first comprehensive multi-omics investigation of QXXY syndrome in IHF patients. By integrating TCM theory with modern systems biology approaches, we aim to unravel the molecular mechanisms underlying QXXY syndrome and its role in IHF progression. Our findings not only advance the understanding of TCM patterns within the framework of modern medicine but also provide a robust foundation for the development of personalized therapeutic strategies for IHF patients.

## Materials and methods

### Study design and participants recruitment

This clinical study was conducted in compliance with the Helsinki Declaration and the Drug Administration Law of the People's Republic of China, and was performed at the First Affiliated Hospital of Henan University of Chinese Medicine from June 2022 to January 2023. The study protocol and informed consent form were approved by the Ethics Committee of the First Affiliated Hospital of Henan University of Chinese Medicine (Approval No: 2021HL-178). All participants were recruited from the cardiology outpatient clinic of the First Affiliated Hospital of Henan University of Chinese Medicine or the surrounding communities in Zhengzhou. All participants signed a written informed consent form agreeing to participate in the study and allowing their data to be publicly disclosed.

### Participants

Patients are eligible for inclusion if they meet the following criteria: 1) Age 40–80 years; 2) Ischemic heart failure patients meeting the 2018 Chinese Guidelines for the diagnosis and treatment of heart failure and the 2022 AHA/ACC/HFSA Guidelines for the management of heart failure [10, 11]; 3) Patients with IHF-QXXY syndrome or IHF-NQXXY syndrome who meet the Guidelines for TCM Diagnosis and Treatment of chronic heart failure (2022) [12]; 4) Left ventricular ejection fraction (LVEF) ≤ 50%, as measured by echocardiography using the modified Simpson method; 5) New York Heart Association (NYHA) Class I to IV; 6) Written informed consent obtained. Healthy adults with normal physical examinations will be included. To minimize diagnostic bias and enhance consistency, all researchers completed standardized training on TCM syndrome differentiation, including a 4-h workshop and certification exam. Subsequently, two independent associate chief TCM physicians, blinded to patients’ biochemical and imaging data, performed syndrome differentiation strictly following the Guidelines for TCM Diagnosis and Treatment of chronic heart failure. Discrepancies in diagnosis were resolved through panel discussion with a third senior TCM expert.

Exclusion criteria include the presence of any of the following: 1) Pulmonary embolism, acute coronary syndrome, or acute cerebrovascular disease; 2) Other heart diseases such as valvular heart disease, severe valve abnormalities, myocardial disease, congenital heart disease, or pulmonary heart disease; 3) Liver and/or kidney dysfunction, malignancies, or autoimmune diseases; 4) Psychosis or substance abuse; 5) Regnancy, planning for pregnancy, or breastfeeding.

### Clinical evaluation index

NT-proBNP levels before and after treatment were measured using a colloidal gold test kit (Getein Biotech, Nanjing, China). LVEF, left ventricular end-diastolic dimension (LVEDD), left ventricular end-diastolic volume (LVEDV), and stroke volume (SV) were measured using a color Doppler ultrasound diagnostic device (GE Vivid E95, USA) employing the two-dimensional Simpson’s method. The 6-min walk distance (6MWD) was assessed by researchers who had completed standardized training. Patient quality of life was assessed using the Minnesota Living with Heart Failure Questionnaire (MLHFQ). Laboratory parameters included blood lipids, blood glucose, coagulation function.

### ELISA assay

Plasma levels of ATP (Beijing Solarbio Science & Technology Co., Ltd., BC0305), Acetyl-CoA (Wuhan Elabscience Biotechnology Co., Ltd., E-EL-0125), ET-1 (Wuhan Elabscience Biotechnology Co., Ltd., E-EL-H0064), NO (Wuhan Elabscience Biotechnology Co., Ltd., E-BC-K035-M), VCAM-1(Wuhan Elabscience Biotechnology Co., Ltd., E-EL-H5587), ICAM-1 (Wuhan Elabscience Biotechnology Co., Ltd., E-EL-H6114), TNF-α (Wuhan Elabscience Biotechnology Co., Ltd., E-EL-H0109), IL-1β (Wuhan Elabscience Biotechnology Co., Ltd., E-EL-H0149), IL-6 (Wuhan Elabscience Biotechnology Co., Ltd., E-EL-H6156), PGI2 (Wuhan Elabscience Biotechnology Co., Ltd., E-EL-0022), TXA2 (Wuhan Elabscience Biotechnology Co., Ltd., E-EL-0057), TAT (Wuhan Elabscience Biotechnology Co., Ltd., E-EL-H1788), MPO (Wuhan Elabscience Biotechnology Co., Ltd., E-BC-K074-M), cell-free DNA (cfDNA, Beijing Solarbio Science & Technology Co., Ltd., D1810), Cit-H3 (Wuhan Colorful Gene Biological Technology Co., Ltd., ELK0742), and NE (Wuhan Elabscience Biotechnology Co., Ltd., E-EL-H1946) were quantified using ELISA kits, following the manufacturer’s instructions.

### RNA-seq-based transcriptomic study

Fasting venous blood samples (2.5 ml) were collected from each subject in the morning using PAXgeneTM tubes (PreAnalytiX, China), gently inverted 8–10 times for thorough mixing, labeled, and gradient-frozen following the manufacturer's instructions. Total RNA was extracted from the blood samples using the PAXgene Blood miRNA Kit (PreAnalytiX, China). RNA concentration and integrity were precisely measured using an Agilent 5400 Bioanalyzer (Agilent Technologies, USA) and a Nanodrop spectrophotometer (BioForge, China). Sequencing was performed on the Illumina NovaSeq 6000 high-throughput platform (Illumina, USA). The raw sequencing data were trimmed, filtered and qualified using FASTX (http://hannonlab.cshl.edu/fastx_toolkit/). Index of the reference genome was built using Hisat2 v2.0.5 and paired-end clean reads were aligned to the reference genome using Hisat2 v2.0.5 [13]. Feature Counts v1.5.0-p3 was used to count the reads numbers mapped to each gene. And then FPKM of each gene was calculated based on the length of the gene and reads count mapped to this gene [14]. DESeq2 was used to estimate the significance of differential genes (DEGs) between any two experimental groups according to the criteria of |log2FC|≥ 1 and adjusted *P < *0.05 (Benjamini–Hochberg correction) [15]. R package “Clusterprofiler” and “DOSE” were used to perform GO, and KEGG enrichment analysis.

### Data-independent-acquisition-based proteomic study

Fasting venous blood samples were collected from each participant in the morning and placed into 2 ml anticoagulant tubes containing EDTA for centrifugation. The supernatant was centrifuged at 3000 × g for 15 min at 4 °C. The fresh supernatant was then collected into Eppendorf tubes, labeled, and stored at − 80 °C. Proteins were extracted and identified following the manufacturer's instructions, using the Proteominer low-abundance protein enrichment kit (Bio-Rad, USA) to deplete high-abundance proteins [16]. Protein concentration in the samples was measured using the Bradford protein assay kit (Biyuntian, China). Enzymatically digested peptides were desalted and analyzed via LC–MS/MS on a Q Exactive HF-X system (Thermo Fisher Scientific, USA) operated in DIA mode. The DIA method involves fragmenting all precursor ions within predefined mass-to-charge (m/z) windows, enabling comprehensive peptide quantification without prior spectral library dependency [17]. Raw MS data were processed using DIA-NN software (v1.8.1) with a spectral library generated from the SWISS-PROT human database (release 2023_10_18) [18]. Search parameters included a precursor mass tolerance of 10 ppm and fragment mass tolerance of 0.02 Da. To ensure data quality, peptides with a false discovery rate (FDR) > 1% were excluded. The DEPs were selected based on criteria of FC > 1.5 or FC < 0.67 and *P < *0.05 [19]. A detailed description of proteomic study methodology can be found in Table S1.

### Targeted metabolomics study

Fasting venous blood samples were collected from each participant in the morning and placed into 2 ml anticoagulant tubes containing EDTA for centrifugation. The supernatant was centrifuged at 3000 × g for 10 min at 4 °C. The fresh supernatant was then collected into Eppendorf tubes, labeled, and stored at − 80 °C. Metabolites were extracted following the manufacturer's protocol and analyzed using an ultra-high performance liquid chromatography-tandem mass spectrometry (UHPLC-MS/MS) system (ExionLC™ AD UHPLC-QTRAP 6500 +, AB SCIEX Corp., USA) operated in multiple reaction monitoring (MRM) mode. Raw data were processed using the metabolomics software metaX, including baseline correction, peak alignment, and normalization to internal standards. Principal component analysis (PCA) and partial least squares-discriminant analysis (PLS-DA) were performed to assess inter-group separation and identify metabolites with variable importance in projection (VIP) scores > 1.0, indicating significant contributions to group discrimination [20]. The statistical significance between the two groups for each metabolite was assessed using a t-test, and FC between the groups were also calculated. Differentially expressed metabolites (DMs) were identified based on the criteria of upregulated metabolites with FC > 1.2, downregulated metabolites with FC < 0.833, and VIP > 1 [21]. Detailed procedures for targeted metabolomics are outlined in Table S2.

### Protein–protein interaction (PPI) network construction and screening candidate biomarkers

Mapping DEGs/DEPs to the STRNG database (https://string-db.org/), with a filter criterion of a minimum required interaction score of 0.4, removing unconnected nodes to establish the PPI network. After downloading PPI analysis results, we input them into the cytoscape software (version 3.10.2) for further optimization. Network topology and node centrality analyses were performed using the cytoHubba plugin. We employed the Maximal Clique Centrality (MCC), Neighborhood Component Centrality (MNC), and Degree algorithms to identify the top 20 key genes/ proteins in the core module.

Next, the “glmnet” function in R was used to perform LASSO regression with tenfold cross-validation (k = 10). The optimal regularization parameter (λ) was selected based on the minimum cross-validation error [22]. For SVM-RFE, implemented via the “e1071” package, randomized feature selection and model optimization were conducted through iterative cross-validation to identify the best feature subset [23]. The RF algorithm (“randomForest” package) generated 500 decision trees through bootstrap aggregation, with variable importance ranked by mean decrease in Gini index [24]. Finally, the intersection of the feature genes/proteins identified by these three machine learning approaches was determined to identify candidate biomarkers associated with IHF-QXXY syndrome. A receiver operating characteristic (ROC) curve was subsequently plotted.

### Joint analysis of transcriptomics, proteomics, and metabolomics

Using the ClueGO and CluePedia plugins of Cytoscape 3.10.2, we analyzed the KEGG pathways and Gene Ontology (GO) enrichment of potential targets based on DEGs/DEPs identified in the QXXY syndrome. This was done to clarify the key biological processes regulated by QXXY syndrome, with pathway analysis set to a significance level of *P* ≤ 0.05. DEGs, DEPs, and DMs related to QXXY syndrome were mapped to the KEGG pathway database using the “Pathview” package in R, allowing us to obtain the shared pathway information. KEGG Mapper was used to highlight the nodes of DEGs, DEPs, and DMs in the enriched pathways, with each node displayed in different colors.

### iPRM quantitative proteomic analysis

Intelligent Parallel Reaction Monitoring (iPRM), an advanced targeted proteomics strategy combining the high selectivity of parallel reaction monitoring (PRM) with dynamic retention time scheduling, was employed to validate candidate proteins [25]. In contrast to conventional PRM, iPRM dynamically adjusts mass spectrometry acquisition parameters based on real-time peptide detection, enhancing sensitivity and quantification accuracy. Samples were retrieved from storage at − 80 °C and processed for protein extraction and quantification using the previously described proteomics protocol. After quality control validation, enzymatic digestion and desalting were performed. Peptide information was acquired in DIA mode, generating raw mass spectrometry data files (.raw). Spectronaut software (v17.0) was used to identify and quantify proteins, selecting unique peptides corresponding to the differential target proteins. The chromatographic method remained consistent with the DIA data acquisition approach. The iPRM list was imported into the mass spectrometry acquisition method settings file and edited to establish the iPRM acquisition method. The acquisition mode utilized was Full MS-PRM.

### Real-time quantitative polymerase chain reaction (RT-qPCR)

The RT-qPCR was conducted using BlazeTaq™ SYBR^®^ Green qPCR mix 2.0 (GeneCopoeia Green, USA), with primer sequences listed in Table S3. A housekeeping gene (β-actin) was used as an endogenous control for normalization. The Relative mRNA expression was calculated using the 2 − ΔΔCt method in a triplicated manner.

### Statistical analysis

Biological data were analyzed using GraphPad Prism version 9.0 (GraphPad Software Inc., CA, USA). Frequency counts and percentages summarized categorical variables, while means and standard deviations (SDs) represented continuous variables. One-way analysis of variance (ANOVA) compared group data. A P-value below 0.05 indicated statistical significance.

## Results

### Demographic factors and baseline characteristics

From June 2022 to January 2023, we recruited 100 eligible participants from the cardiovascular outpatient clinic of the First Affiliated Hospital of Henan University of Chinese Medicine and surrounding communities, comprising 40 IHF patients with QXXY Syndrome (IHF-QXXY), 40 with NQXXY Syndrome (IHF-NQXXY), and 20 healthy controls (HC). The demographic and clinical biochemical characteristics of the participants are presented in Table 1. Age, sex, DBP, and BMI did not differ significantly among the IHF-QXXY, IHF-NQXXY, and HC groups (*P > *0.05). However, significant differences were observed in SBP, blood lipid and glucose levels, as well as PT and APTT, reflecting glucose-lipid metabolism disorders and coagulation abnormalities in IHF patients. The IHF-QXXY and IHF-NQXXY groups showed no statistically significant differences in age, sex, DBP, BMI, history of hypertension, hyperlipidemia, diabetes, or laboratory findings. Notably, compared with the IHF-NQXXY group, the IHF-QXXY group had a greater proportion of patients with NT-proBNP levels below 1000 pg/ml and NYHA functional class I–II. Furthermore, the IHF-QXXY group demonstrated a higher LVEF (41.00% vs. 35.28%, *P = *0.001), longer 6WMD (371.37 m vs. 312.23 m, *P = *0.014), and lower MLHFQ scores (44.98 vs. 52.93, *P = *0.019). These differences indicate that QXXY Syndrome patients are in a relatively stable phase of IHF, whereas those with NQXXY Syndrome may be experiencing disease progression (Table 2).

**Table 1:** Baseline demographic and biochemical characteristics

	QXXYZ (n = 40)	FQXXYZ (n = 40)	HP (n = 20)
Age, years	67.68 (7.85)	67.28 (8.03)	64.95 (3.18)
Female, n (%)	6 (15.00%)	12 (30.00%)	7 (35.00%)
Seated SBP, mmHg	133.75 ± 21.89	124.33 ± 18.70	118.55 ± 17.80
Seated DBP, mmHg	74.83 ± 14.36	69.35 ± 11.24	74.7 ± 10.56
BMI, kg/cm^2^	24.15 ± 3.77	23.34 ± 2.96	23.78 ± 3.65
Hypertension, n (%)	18 (45.00%)	17 (42.5%)	0 (0.00%)
Diabetes mellitus, n (%)	8 (20.00%)	11 (27.5%)	0 (0.00%)
Hyperlipidemia, n (%)	25 (62.50%)	26 (65.00%)	0 (0.00%)
Use sacubitril valsartan, n (%)	14 (35.50%)	13 (32.50%)	0 (0.00%)
Biochemical profile			
TC, mmol/L	3.72 (0.98)	3.81 (0.89)	4.42 (0.64)
TG, mmol/L	1.39 (0.58)	1.74 (1.06)	1.10 (0.35)
HDL-C, mmol/L	1.20 (0.27)	1.15 (0.28)	1.29 (0.20)
LDL-C, mmol/L	2.12 (0.72)	2.18 (0.65)	2.95 (0.53)
GLU, mmol/L	6.5 (2.46)	6.3 (2.14)	5.04 (0.48)
PT, s	12.27 (1.26)	11.89 (1.96)	10.74 (0.55)
APTT, s	28.25 (3.55)	29.29 (3.74)	31.69 (2.73)
TT, s	15.36 (1.89)	15.05 (1.51)	15.31 (0.89)
FIB, g/L	3.21 (0.88)	3.41 (1.15)	3.32 (0.55)

Data are n (%) or mean (SD).

*SBP* systolic blood pressure, *DBP* diastolic blood pressure, *BMI* body mass index, *TC* total cholesterol, *TG* triglycerides, *HDL-C* high-density lipoprotein cholesterol, *LDL-C* low-density lipoprotein cholesterol, *GLU* glucose, *PT* prothrombin time, *APTT* activated partial thromboplastin time, *TT* thrombin time, *FIB* fibrinogen

**Table 2:** The difference of cardiac function indexes between IHF-QXXY group and IHF-NQXXY group

	IHF-QXXY (n = 40)	IHF-NQXXY (n = 40)	P-value
NYHA classification			0.086
I ~ II, n (%)	20 (50.00%)	13 (32.50%)	
III ~ IV, n (%)	20 (50.00%)	27 (67.50%)	
NT-proBNP (pg/ml)			0.044
< 1000 pg/ml, n (%)	25 (62.5%)	15 (47.50%)	
≥ 1000 pg/ml, n (%)	15 (37.50%)	25 (52.50%)	
LVEF (%)	41.00 ± 6.35	35.28 ± 7.87	0.001
LVEDD (mm)	57.73 ± 6.77	58.55 ± 8.71	0.638
LVEDV (ml)	143.41 ± 62.81	147.10 ± 60.09	0.789
SV (ml)	58.03 ± 22.71	50.70 ± 18.68	0.119
6MWD (m)	371.37 ± 77.22	312.23 ± 126.04	0.014
MLHFQ	44.98 ± 15.93	52.93 ± 16.37	0.019

Data are n (%) or mean (SD)

*NYHA* New York Heart Association, *NT-proBNP* N-terminal pro-B-type natriuretic peptide, *LVEF* left ventricular ejection fraction, *LVEDD* left ventricular end-diastolic diameter, *LVEDV* left ventricular end-diastolic volume, *SV* stroke volume, *6 MWD* 6-min walking distance, *MLHFQ* Minnesota living with heart failure questionnaire

### Biological characteristics of IHF-QXXY syndrome

**Fig. 1:**
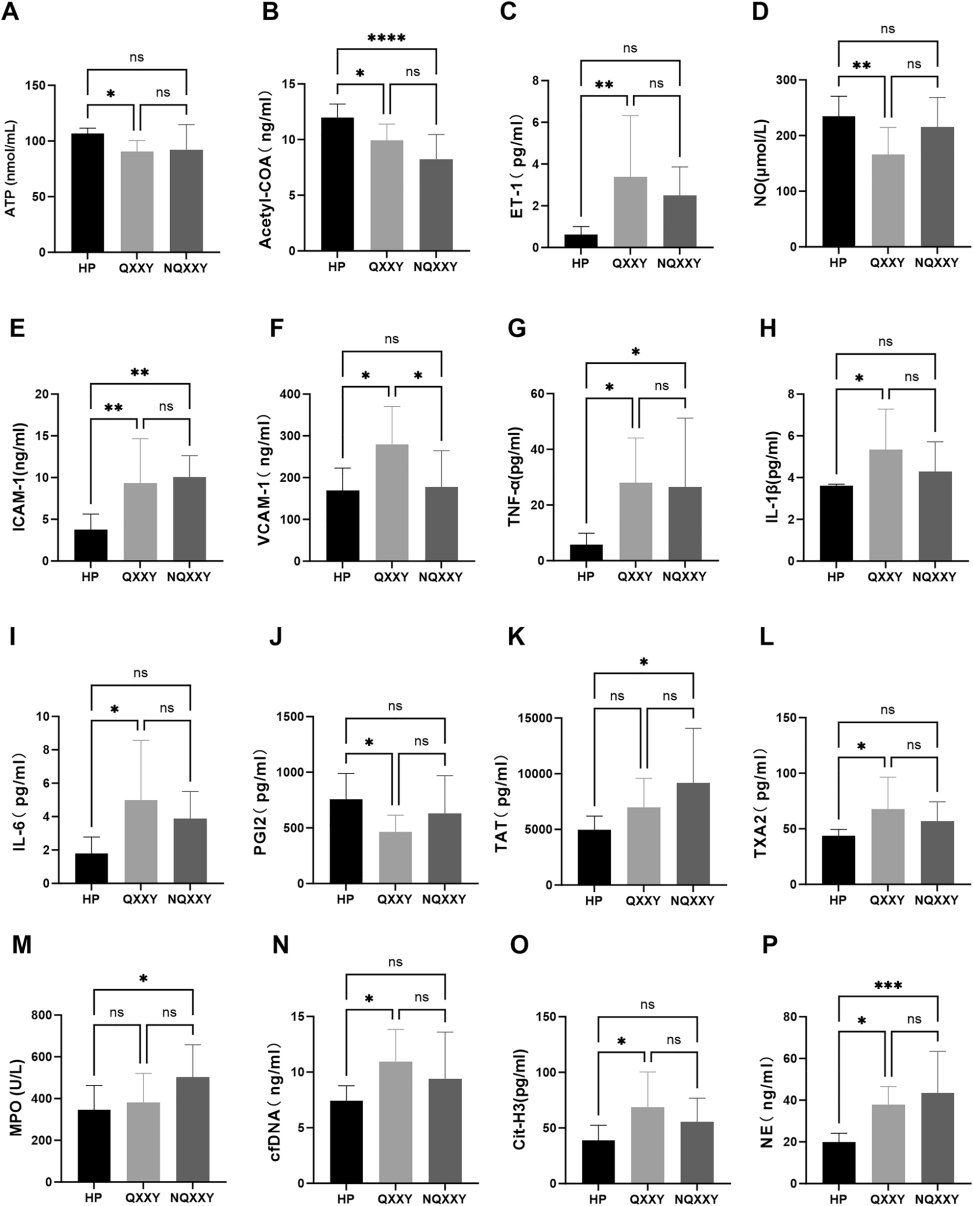
Biological characteristics of IHF-QXXY syndrome. **A** The level of ATP in each group was detected by ELISA kit. **B** The level of Acetyl-CoA in each group was detected by ELISA kit. **C** The level of ET-1 in each group was detected by ELISA kit. **D** The level of NO in each group was detected by ELISA kit. **E** The level of ICAM-1 in each group was detected by ELISA kit. **F** The level of VCAM-1 in each group was detected by ELISA kit. **G** The level of TNF-α in each group was detected by ELISA kit. **H** The level of IL-1β in each group was detected by ELISA kit. **I** The level of IL-6 in each group was detected by ELISA kit. **J** The level of PGI2 in each group was detected by ELISA kit. **K** The level of TAT in each group was detected by ELISA kit. **L** The level of TXA2 in each group was detected by ELISA kit. **M** The level of MPO in each group was detected by ELISA kit. **N** The level of cfDNA in each group was detected by ELISA kit. **O** The level of Cit-H3 in each group was detected by ELISA kit. **P** The level of NE in each group was detected by ELISA kit. *P < 0.05; ** *P < *0.01; *** *P < *0.001; **** *P < *0.0001

To elucidate the biological characteristics of IHF-QXXY syndrome, we assessed 16 indicators associated with energy metabolism, endothelial function, inflammation, coagulation, and neutrophil extracellular traps (NETs) formation. The assessed indicators included ATP, acetyl-CoA, ET-1, NO, ICAM-1, VCAM-1, TNF-α, IL-1β, IL-6, PGI2, TAT, TXA2, MPO, cfDNA, Cit-H3, and NE. Our results showed a significant decrease in energy metabolism indicators in IHF-QXXY syndrome patients (Fig. 1A–B), with ATP levels decreasing by an average of 16.36 nmol/ml (*P < *0.05) and acetyl-CoA by 2.038 ng/ml (*P < *0.05) compared to the HC group. Compared to IHF-FXXY syndrome, ATP and acetyl-CoA levels showed no significant differences (*P > *0.05). Endothelial function indicators were significantly altered in IHF-QXXY syndrome patients (Fig. 1C–D), with ET-1 levels increasing by 2.764 pg/ml (*P < *0.01) and NO levels decreasing by 68.10 μmol/L (*P < *0.01) compared to the HC group. ET-1 and NO levels did not significantly differ between IHF-QXXY and IHF-FXXY syndrome (*P > *0.05). Patients with IHF-QXXY syndrome exhibited significantly elevated inflammatory response markers (Fig. 1E–I). Compared to the HC group, ICAM-1 levels increased by 5.541 ng/ml (*P = *0.005), VCAM-1 by 110.6 ng/ml (*P < *0.05), TNF-α by 22.22 pg/ml (*P < *0.05), IL-1β by 1.731 pg/ml (*P < *0.05), and IL-6 by 3.193 pg/ml (*P < *0.05). Compared to the IHF-NQXXY syndrome group, VCAM-1 levels were further elevated (*P < *0.05), whereas other inflammatory markers showed no significant differences (*P > *0.05). These findings indicate that patients with IHF QXXY syndrome experience a marked chronic inflammatory response. Additionally, patients with IHF-QXXY syndrome displayed abnormal coagulation function markers (Fig. 1J–L). Compared to the healthy group, PGI2 levels decreased by 294.2 pg/ml (*P < *0.05), TXA2 levels increased by 24.05 pg/ml (*P < *0.05), and TAT levels showed an upward trend. Patients with IHF-QXXY syndrome exhibited significantly elevated NETs-related markers (Fig. 1M–P). Compared to the HC group, cfDNA concentration increased by an average of 3.533 ng/ml (*P < *0.05), Cit-H3 by 30.02 pg/ml (*P < *0.05), NE by 17.99 ng/ml (*P < *0.05), and MPO showed a slight upward trend (*P < *0.05). These findings suggest that NETs formation plays a role in the progression of IHF-QXXY syndrome.

### Transcriptomic characteristics of IHF-QXXY syndrome

**Fig. 2:**
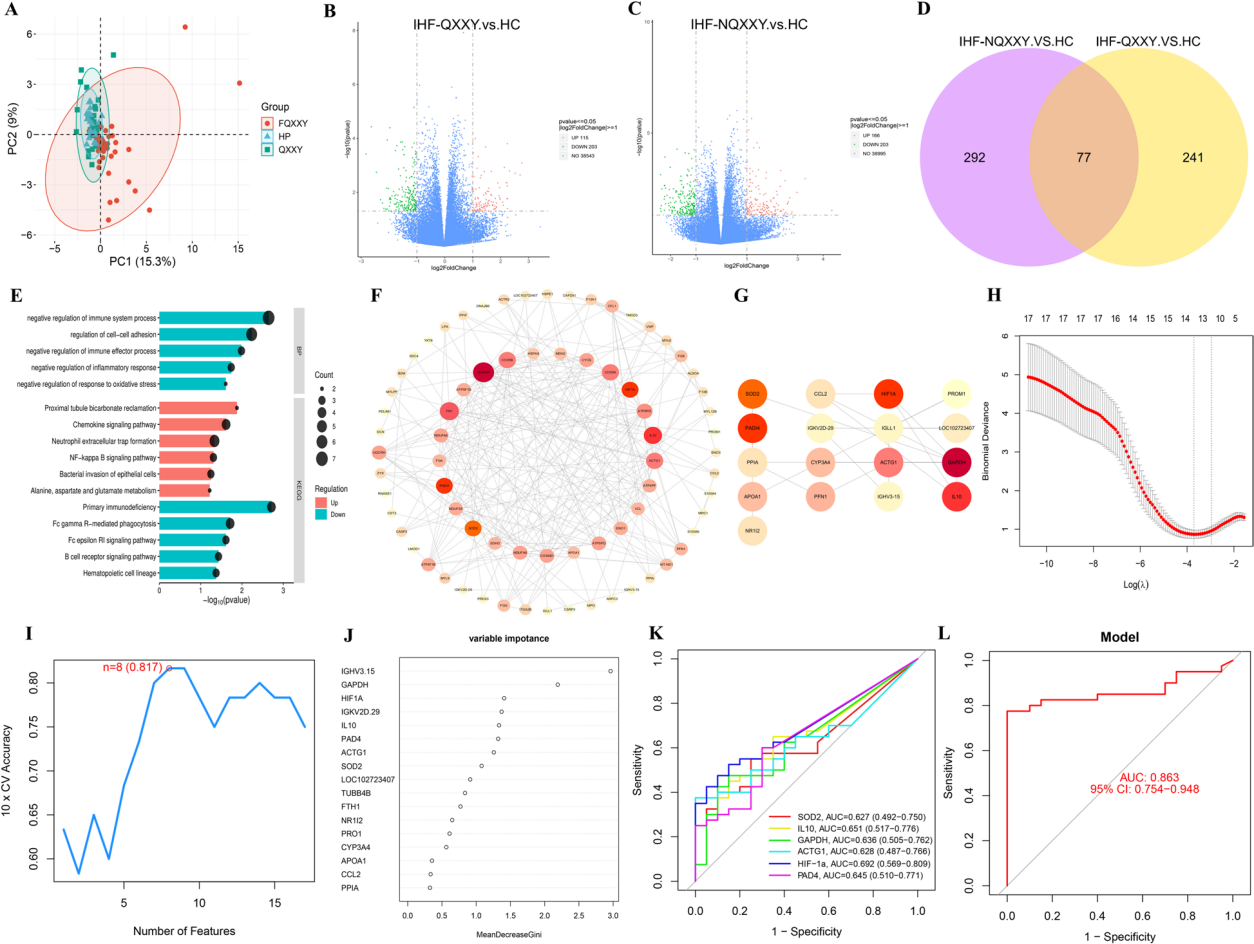
Transcriptomic characteristics of IHF-QXXY syndrome. **A** Classification results of HC, IHF-QXXY, and IHF-NQXXY groups based on PCA score plot. **B** The volcano plots of DEGs between IHF-QXXY and HC group. **C** The volcano plots of DEGs between IHF-NQXXY and HC group. **D** Venn diagram of specific DEGs to IHF-QXXY syndrome. **E** Pathway enrichment of specific DEGs to IHF-QXXY syndrome. **F** PPI network analysis of specific DEGs to IHF-QXXY syndrome. **G** The top 17 nodes of the PPI network identified by the MCC, MNC, and Degree algorithms. **H** LASSO analysis screening of feature genes. **I** SVM-RFE algorithm screening of feature genes. **J** RF algorithm screening of feature genes. **K** ROC curve of HIF-1α, IL10, PAD4, ACTG1, SOD2, and GAPDH. **L** Combined diagnostic ROC curves of feature genes

Blood transcriptomes from the HC, IHF-QXXY, and IHF-NQXXY groups were sequenced using the Illumina high-throughput platform. PCA demonstrated clear separations among the HC, IHF-QXXY, and IHF-NQXXY groups (Fig. 2A). The DESeq2 software was used for intergroup analysis of DEGs(|log2FC|≥ 1, *P < *0.05). The IHF-QXXY vs. HC comparison revealed 318 DEGs, with 115 upregulated and 203 downregulated genes (Fig. 2B). The IHF-NQXXY vs. HC comparison identified 369 DEGs, with 166 upregulated and 203 downregulated genes (Fig. 2C). Venn diagram analysis (Fig. 2D) identified 241 DEGs unique to QXXY syndrome, comprising 121 upregulated and 120 downregulated mRNAs. Using clusterProfiler (version 4.12.0), pathway enrichment analysis of QXXY syndrome-specific DEGs revealed significant enrichment in immune-inflammatory pathways, including NETs Formation, Chemokine Signaling, NF-kappa B Signaling, and B Cell Receptor Signaling (Fig. 2E). To explore key genes in QXXY syndrome, a gene–gene interaction network was constructed and optimized in Cytoscape using STRING data, comprising 98 nodes and 239 edges (Fig. 2F). CytoHubba analysis integrating MCC, MNC, and Degree algorithms identified 17 hub DEGs with high centrality and betweenness (Fig. 2G). Machine learning analysis of the 17 hub DEGs identified 13 feature genes by LASSO, 8 by SVM-RFE, and 8 by RF (Fig. 2H–J). The intersection of these methods revealed six candidate biomarkers (HIF-1α, IL10, PAD4, ACTG1, SOD2, and GAPDH) (Table S4). ROC analysis showed that the AUC values for HIF-1α, IL10, PAD4, ACTG1, SOD2, and GAPDH ranged from 0.627 to 0.693 (Fig. 2K). The combined diagnostic AUC reached 0.863 (95% CI: 0.754–0.948), suggesting strong diagnostic potential and reliability (Fig. 2L).

### Proteomic characteristics of IHF-QXXY syndrome

**Fig. 3:**
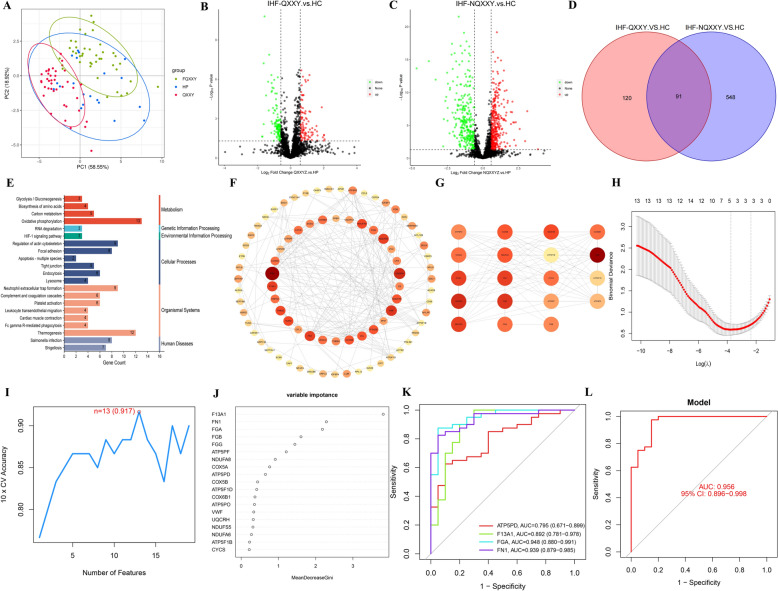
Proteomic characteristics of IHF-QXXY syndrome. **A** Classification results of HC, IHF-QXXY, and IHF-NQXXY groups based on PLS-DA score plot. **B** The volcano plots of DEPs between IHF-QXXY and HC group. **C** The volcano plots of DEPs between IHF-NQXXY and HC group. **D** Venn diagram of specific DPGs to IHF-QXXY syndrome. **E** Pathway enrichment of specific DEPs to IHF-QXXY syndrome. **F** PPI network analysis of specific DEPs to IHF-QXXY syndrome. **G** The top 19 nodes of the PPI network identified by the MCC, MNC, and Degree algorithms. **H** LASSO analysis screening of feature proteins. **I** SVM-RFE algorithm screening of feature proteins. **J** RF algorithm screening of feature proteins. **K** ROC curve of FGA, FN1, F13A1, and ATP5PF. **L** Combined diagnostic ROC curves of feature proteins

Next, we conducted DIA-based proteomics analysis. The results of PLS-DA revealed clear sample dispersion between different groups, while samples within the same group clustered together (Fig. 3A). A total of 759 DEPs were obtained in IHF-QXXY vs. HC group and IHF-NQXXY vs. HC group. A total of 211 DEPs were identified between the QXXY and HC groups, with 100 upregulated and 111 downregulated (Fig. 3B). Between the NQXXY and HC groups, 639 DEPs were identified, including 356 upregulated and 283 downregulated (Fig. 3C). A Venn diagram intersection analysis identified 120 differential proteins specific to QXXY syndrome, with 41 upregulated DEPs and 79 downregulated DEPs (Fig. 3D). KEGG pathway enrichment analysis was conducted on the specific DEPs of QXXY syndrome to identify the key biochemical and signaling pathways involved. The results revealed that these DEPs were primarily associated with NETs, complement and coagulation cascades, platelet activation, glycolysis/gluconeogenesis, and the HIF-1 signaling pathway (Fig. 3E). The PPI network of specific DEPs for QXXY syndrome was constructed using Cytoscape, as shown in Fig. 3F, with 105 nodes and 239 edges. Using multiple algorithms based on the CytoHubba plugin, an intersection was performed, identifying 19 DEPs with high centrality and betweenness centrality (Fig. 3G). Additionally, LASSO, SVM-RFE, and RF algorithms identified 5, 13, and 7 feature proteins, respectively (Fig. 3H–J, Table S5). The intersection of the feature proteins from these algorithms is presented in a Venn diagram, identifying candidate biomarkers FGA, FN1, F13A1, and ATP5PF. ROC analysis showed that the AUC values for FGA, FN1, F13A1, and ATP5PF were 0.948, 0.939, 0.892, and 0.787, respectively Fig. 3K). The combined diagnostic performance of these biomarkers yielded an AUC of 0.956 (95% CI: 0.896–0.998), underscoring their diagnostic accuracy and reliability in assessing QXXY syndrome (Fig. 3L).

### Metabolomic characteristics of IHF-QXXY syndrome

**Fig. 4:**
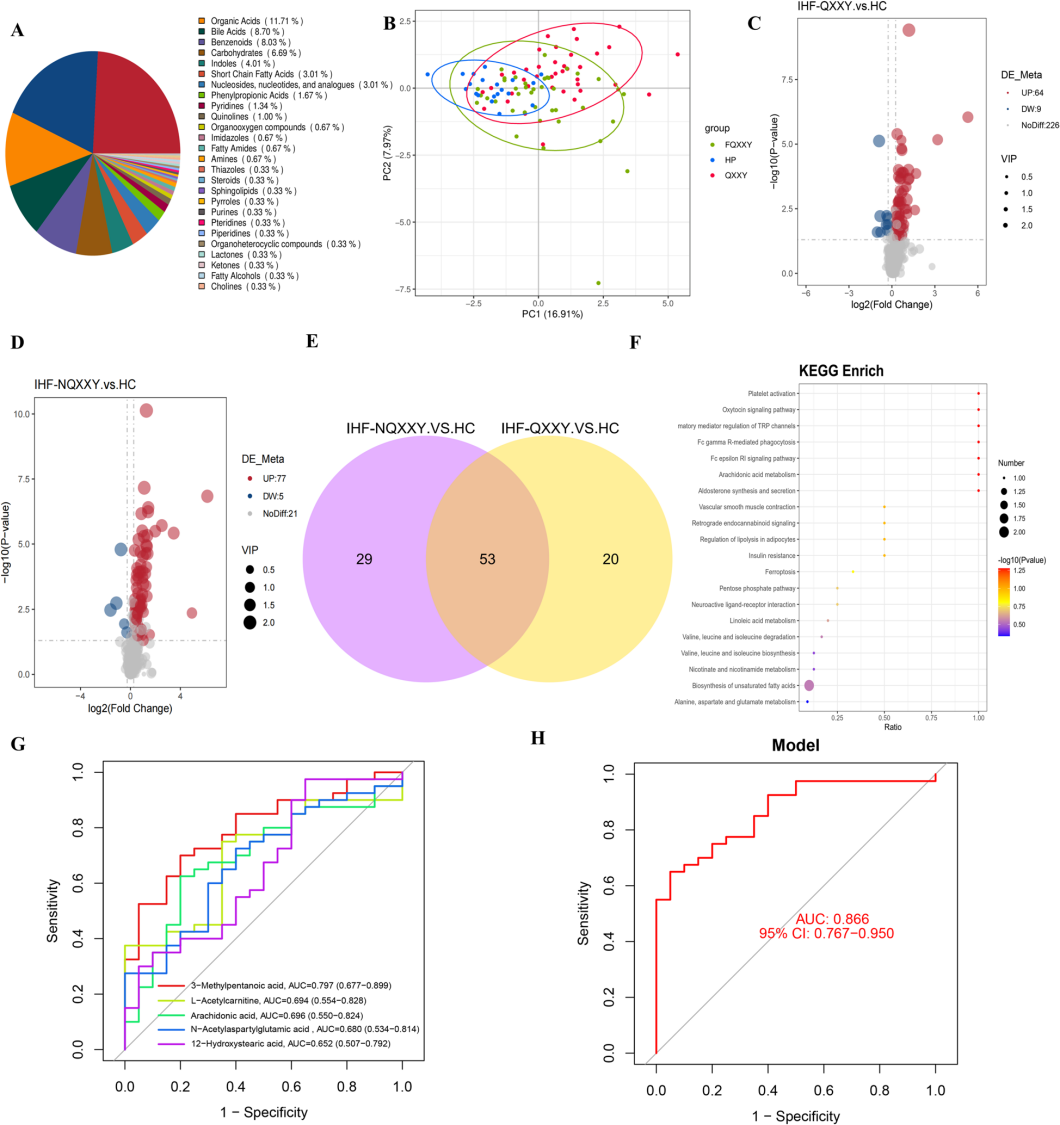
Metabolomic characteristics of IHF-QXXY syndrome. **A** Pie chart of identified metabolites. **B** PLS-DA score plot of the HC, IHF-QXXY, and IHF-NQXXY groups. **C** The volcano plots of DMs between IHF-QXXY and HC group. **D** The volcano plots of DMs between IHF-NQXXY and HC group. **E** Venn diagram of specific DMs to IHF-QXXY syndrome. **F** Pathway enrichment of specific DMs to IHF-QXXY syndrome. **G** ROC curve of 3-methylpentanoic acid, arachidonic acid, N-acetylaspartylglutamic acid, L-acetylcarnitine, and 12-hydroxystearic acid. **H** Combined diagnostic ROC curves of Key metabolites

Targeted metabolite identification and quantification were conducted on blood samples from the HC, IHF-QXXY, and IHF-NQXXY groups using multiple reaction monitoring. In total, 299 metabolites were identified and classified into 26 categories, with the predominant groups including organic acids (11.71%), bile acids (8.70%), benzenoids (8.03%), carbohydrates (6.69%), indoles (4.01%), short-chain fatty acids (4.01%), nucleosides, nucleotides, and analogues (3.01%), phenylpropionic acids (1.67%), pyridines (1.34%), quinolines (1.00%), and fatty amides (0.67%), as shown in Fig. 4A. Using the PLS-DA model, we established the relationship between metabolite expression levels and sample groups, demonstrating significant differences among the HP, QXXY, and NQXXY groups (Fig. 4B). Using the criteria of VIP > 1, *P < *0.05, and FC > 1.2 or FC < 0.83, we identified 150 endogenous DMs in plasma, including 73 DMs between the QXXY and HC groups, 82 DMs between the NQXXY and HC groups (Fig. 4C, D), and 20 DMs specific to QXXY syndrome (Fig. 4E). Functional enrichment analysis (Fig. 4F) indicated that the DMs specific to QXXY syndrome were predominantly enriched in pathways related to arachidonic acid metabolism, platelet activation, biosynthesis of unsaturated fatty acids, alanine, aspartate, and glutamate metabolism, as well as valine, leucine, and isoleucine degradation. ROC diagnostic analysis demonstrated that the AUC values for 3-methylpentanoic acid, arachidonic acid, N-acetylaspartylglutamic acid (NAAG), L-acetylcarnitine, and 12-hydroxystearic acid were 0.798, 0.696, 0.649, 0.694, and 0.653, respectively (Fig. 4G). When combined, these metabolites exhibited an enhanced diagnostic performance with an AUC of 0.866 (95% CI: 0.767–0.950), suggesting their potential as key metabolic markers for QXXY syndrome (Fig. 4H).

### Integrated analysis of DEGs, DEPs, and DMs

**Fig. 5:**
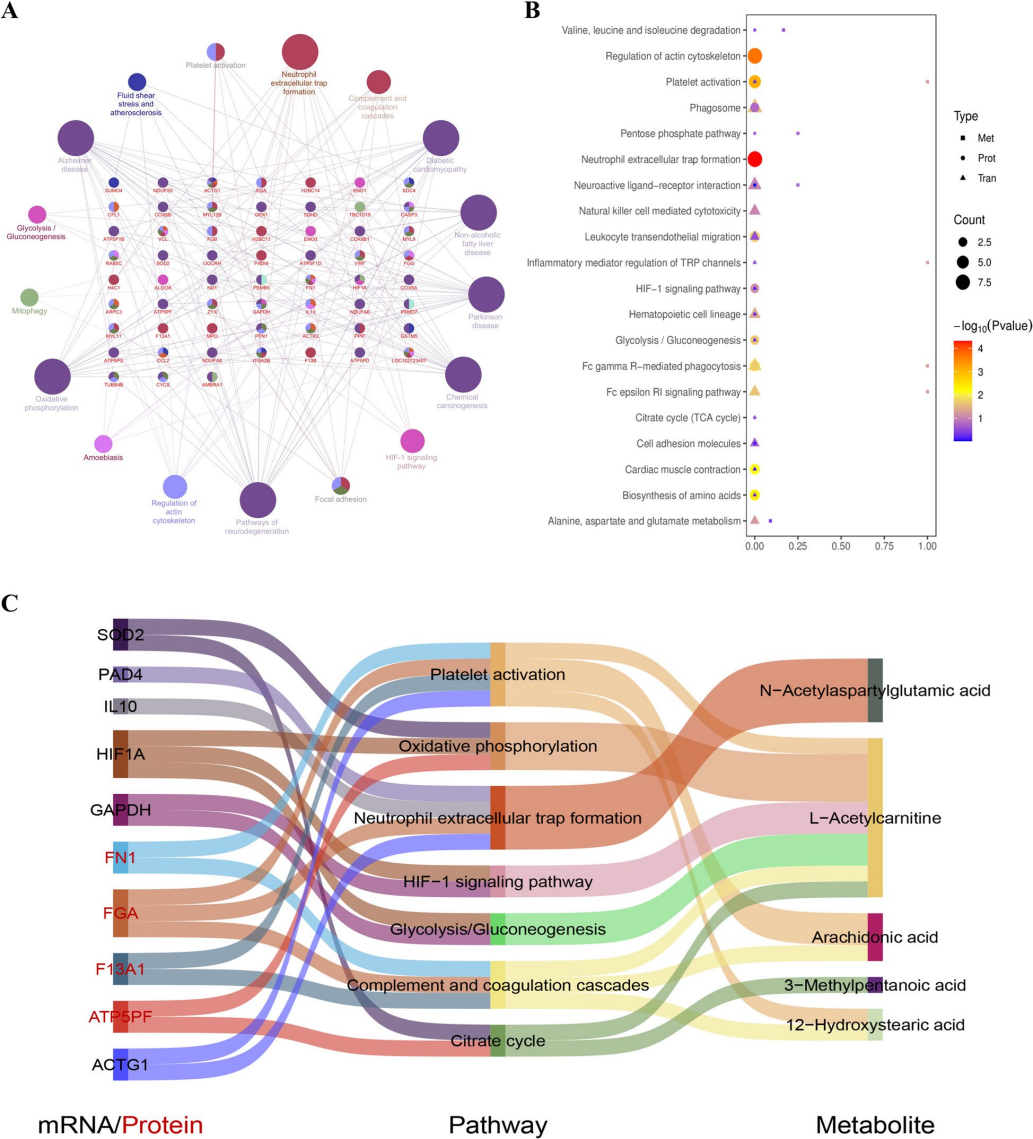
Integrated analysis of multi-omics experiments. **A** ClueGO analysis of KEGG pathway enrichment. **B** Enrichment analysis of KEGG pathway shared by DEGs, DEPs, and DMs. **C** IHF-QXXY syndrome “gene-protein-metabolite” network

The biological foundation of QXXY syndrome was elucidated through the integration of transcriptomics, proteomics, and metabolomics data. Initially, KEGG pathway enrichment analysis was performed using ClueGO and CluePedia, based on RNA-seq and proteomics data, with statistical significance defined as *P < *0.05. KEGG enrichment analysis showed that the pathways that had significant influence were Neutrophil extracellular trap formation, Platelet activation, Complement and coagulation cascades, HIF-1 signaling pathway, Glycolysis/Gluconeogenesis, and oxidative phosphorylation (Fig. 5A). Within the KEGG pathway network, we identified candidate biomarkers such as HIF-1α, IL10, PAD4, ACTG1, SOD2, GAPDH, FGA, FN1, F13A1, and ATP5PF, all of which interact with other genes. Subsequently, the DEGs, DEPs, and DMs associated with QXXY syndrome were mapped to the KEGG pathway database to uncover shared pathway information and identify key biochemical and signaling pathways. Results revealed that Platelet Activation, NETs, HIF-1 Signaling, Citrate Cycle, Valine, Leucine, and Isoleucine Degradation, and Alanine, Aspartate, and Glutamate Metabolism were the most enriched pathways in DEGs, DEPs, and DMs (Fig. 5B). Additionally, candidate biomarkers played pivotal roles within these pathways. Furthermore, we found that DEGs, and DEPs were predominantly upregulated in the HIF-1 Signaling Pathway, Glycolysis/Gluconeogenesis, and NETs, whereas they were mostly downregulated in Platelet Activation. Due to energy metabolism disorders, Valine, Leucine, and Isoleucine Degradation as well as Alanine, Aspartate, and Glutamate Metabolism were upregulated, whereas the Citrate Cycle exhibited a downregulation trend. Therefore, we constructed a “gene-protein-pathway-metabolite” network for QXXY syndrome. The multi-omics network of QXXY syndrome comprises six mRNAs, four proteins, five metabolites, and seven pathways (Fig. 5C). We propose that QXXY syndrome’s biological basis is closely linked to energy metabolism dysregulation, immune-inflammatory responses, and coagulation abnormalities mediated by pathways such as NETs, Platelet Activation, Complement and Coagulation Cascades, HIF-1 Signaling, Glycolysis/Gluconeogenesis, Citrate Cycle, and Oxidative Phosphorylation.

### RT-qPCR validation

**Fig. 6:**
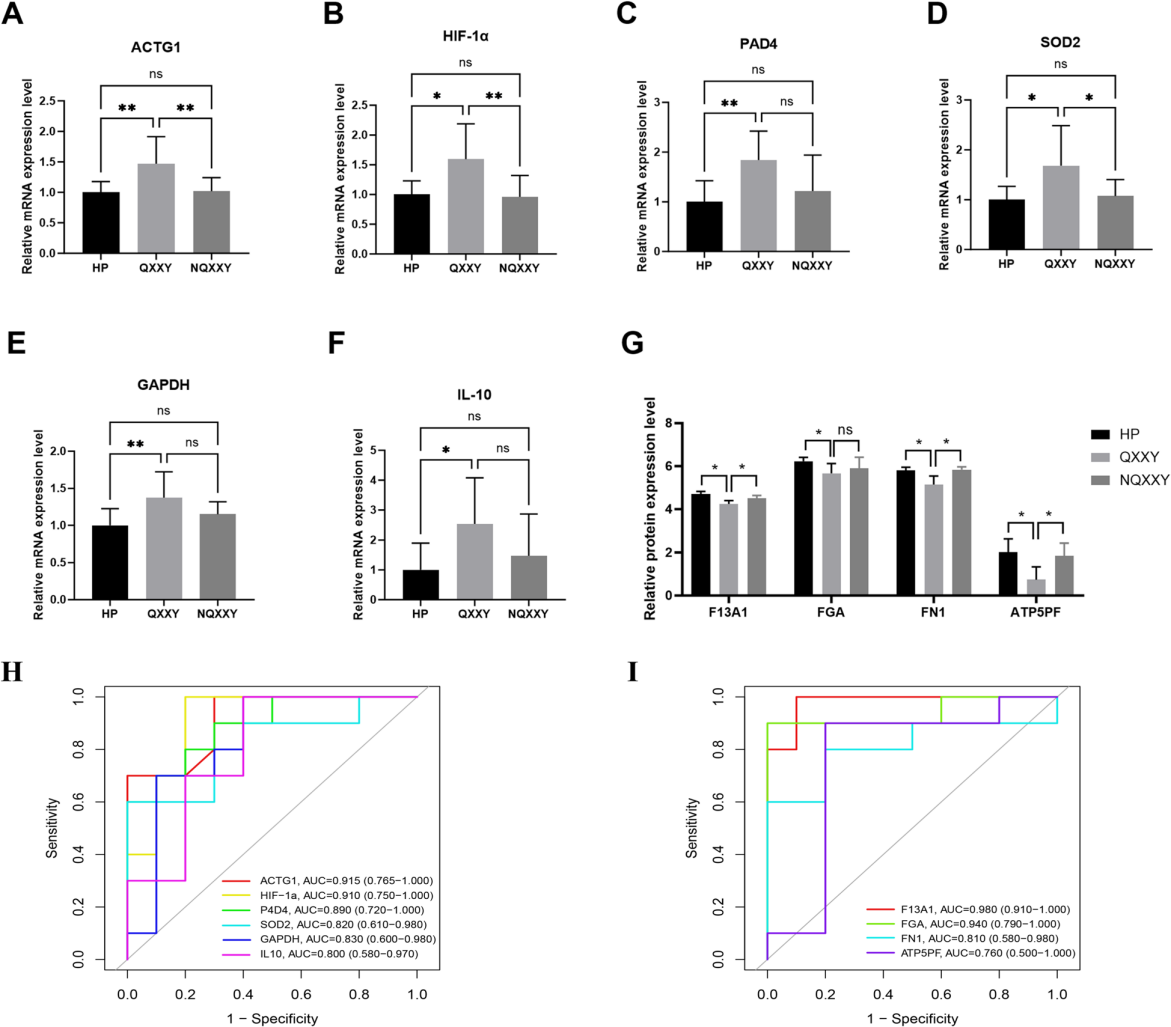
The expression of candidate markers was verified by RT-qPCR and iPRM. **A** The mRNA expression of ACTG1. **B** The mRNA expression of HIF-1α. **C** The mRNA expression of PAD4. **D** The mRNA expression of SOD2. **E** The mRNA expression of GAPDH. **F** The mRNA expression of IL-10. **G** iPRM validation of key DEPs. **H** Validated ROC curves of HIF-1α, IL10, PAD4, ACTG1, SOD2, and GAPDH. **I** Validated ROC curve of FGA, FN1, F13A1, and ATP5PF. ^*^P < 0.05; ^**^ *P < *0.01

We further validated the expression of the feature genes identified in transcriptomics. We randomly selected blood samples from 10 patients in each of the HP, QXXY, and FQXXY groups and used RT-qPCR to validate the expression levels of HIF-1α, IL10, PAD4, ACTG1, SOD2, and GAPDH. The results showed that the expression levels of HIF-1α, IL10, PAD4, ACTG1, SOD2, and GAPDH were higher in heart failure patients with Qi deficiency and blood stasis syndrome compared to the HP group. The ROC curve was used to assess HIF-1α, IL10, PAD4, ACTG1, SOD2, and GAPDH (Fig. 6A–F). The AUC values for the molecules were as follows: ACTG1 [0.915, 95% CI (0.765, 1.000)] > HIF-1α [0.910, 95% CI (0.750, 1.000)] > PAD4 [0.890, 95% CI (0.720, 1.000)] > SOD2 [0.820, 95% CI (0.610, 0.980)] > GAPDH [0.830, 95% CI (0.600, 0.980)] > IL10 [0.800, 95% CI (0.580, 0.970)] (Fig. 6H).

### iPRM validation

To further validate the feature proteins identified in the discovery phase, iPRM-targeted proteomics was used to verify the feature proteins FGA, FN1, F13A1, and ATP5PF4 in QXXY syndrome. Among the detected proteins, the key target proteins FGA, FN1, and F13A1 in the platelet activation pathway were significantly downregulated, while the key protein ATP5PF4 in the energy metabolism pathway also showed significant downregulation, as shown in Fig. 6G. ROC curve analysis was conducted to evaluate the diagnostic performance of FGA, FN1, F13A1, and ATP5PF4. The AUC values for these proteins were as follows: F13A1 [0.980, 95% CI (0.910, 1.000)] > FGA [0.940, 95% CI (0.790, 1.000)] > FN1 [0.810, 95% CI (0.580, 0.980)] > ATP5PF4 [0.760, 95% CI (0.500, 1.000)], demonstrating high diagnostic performance and further validating their reliability as feature proteins for IHF-QXXY syndrome (Fig. 6I).

## Discussion

This study is the first to systematically investigate the biological basis of QXXY syndrome in IHF patients by integrating clinical samples, multi-omics analysis, and machine learning approaches. Through transcriptomic, proteomic, and metabolomic analyses, we established a comprehensive “gene-protein-metabolite” network, encompassing six mRNAs, four proteins, and five metabolites, which delineates the distinct molecular characteristics of IHF-QXXY syndrome. Multi-omics association analysis and functional annotation indicate that the biological underpinnings of Qi deficiency and blood stasis syndrome are linked to disruptions in energy metabolism, chronic inflammatory responses, and coagulation dysfunction, mediated through pathways including NETs, platelet activation, complement and coagulation cascades, HIF-1 signaling, glycolysis/gluconeogenesis, the citrate cycle, and oxidative phosphorylation. These findings suggest that QXXY syndrome, as a subtype of IHF, may have unique molecular changes.

### Energy metabolism dysregulation in QXXY syndrome

Our study results indicate significant metabolic abnormalities in IHF-QXXY syndrome patients, characterized by reduced cardiac function, decreased exercise tolerance, and a marked decline in ATP and Acetyl-CoA levels. This observation aligns with the TCM theory of “Qi deficiency,” which suggests impairments in energy production and utilization. The heart, with its high energy demands, demonstrates exceptional metabolic adaptability, efficiently utilizing diverse energy substrates including fatty acids, glucose, amino acids, lactate, and ketone bodies to meet fluctuating ATP requirements [26]. This adaptability is orchestrated by a complex and interactive metabolic network that undergoes dynamic remodeling in chronic pathological states. However, during IHF progression, this metabolic adaptability is compromised, evidenced by altered substrate utilization, impaired oxidative phosphorylation, mitochondrial dysfunction, and suppression of key metabolic enzymes [27]. Our study further corroborates this hypothesis by demonstrating the downregulation of key energy metabolism pathways, including the citric acid cycle and oxidative phosphorylation, alongside the activation of glycolysis and alanine, aspartate, and glutamate metabolism. Additionally, ROC curve analysis identified several key metabolites: 3-Methylpentanoic acid, Arachidonic acid, NAAG, L-Acetylcarnitine, and 12-Hydroxystearic acid, which hold significant diagnostic potential for QXXY syndrome. Notably, L-Acetylcarnitine, a key intermediate in fatty acid metabolism, exhibited reduced levels, suggesting impaired mitochondrial fatty acid oxidation, a hallmark of the energy deficiencies frequently observed in IHF patients [28]. Likewise, elevated 12-Hydroxystearic acid levels, a product of lipid metabolism, indicate a crucial role of lipid metabolic disturbances in QXXY syndrome [29]. The downregulation of ATP5PF, a pivotal protein in mitochondrial ATP synthesis, further underscores the critical role of mitochondrial dysfunction in QXXY syndrome [30]. As the powerhouse of cellular energy production, mitochondrial dysfunction leads to diminished ATP synthesis, manifesting as fatigue and reduced exercise capacity. These findings align with previous studies, including those by Yuehuai Hu et al., who reported a widespread downregulation of mitochondrial function-related proteins in heart failure patients, leading to metabolic disruptions and cardiac impairment [31, 32]. Moreover, HIF-1α upregulation likely serves as an adaptive mechanism to counteract energy shortages. Under hypoxic conditions, HIF-1α remains stabilized, enhancing anaerobic metabolism through the regulation of glycolysis-related genes (e.g., GAPDH) to offset mitochondrial oxidative phosphorylation deficits [33].

### Chronic inflammatory responses in QXXY syndrome

Chronic inflammation is a hallmark of QXXY syndrome, as evidenced by increased levels of pro-inflammatory cytokines (e.g., TNF-α, IL-1β, IL-6) and adhesion molecules (e.g., ICAM-1, VCAM-1). Since 1990, studies have confirmed elevated levels of various inflammatory mediators, including TNF-α, IL-6, IL-1β, IL-18, and immune antigens, in the plasma of heart failure patients. These findings support the “cytokine hypothesis,” which posits that inflammation plays a crucial role in adverse cardiac remodeling [34]. Notably, increased circulating cytokine levels correlate with heart failure severity and serve as prognostic indicators [35]. The enrichment of the NF-κB and chemokine signaling pathways further highlights the critical role of immune dysregulation in QXXY syndrome. PAD4 is a crucial post-translational modification enzyme that facilitates chromatin decondensation and DNA release through histone citrullination, acting as a central regulator of NET formation [36]. Intriguingly, our transcriptomic analysis reveals a significant upregulation of PAD4, indicating a potential pivotal role of NETs in the inflammatory response of QXXY syndrome. This finding aligns with recent research [37, 38], demonstrating the critical involvement of NETs in the pathogenesis of cardiovascular diseases, including IHF. Histones and DNA fragments within NETs function as damage-associated molecular patterns (DAMPs), activating the innate immune system and stimulating monocytes and macrophages to release substantial pro-inflammatory cytokines. This inflammatory amplification loop further exacerbates myocardial inflammation and tissue injury [39]. Increased levels of NET-associated biomarkers (e.g., Cit-H3, NE) further substantiate this hypothesis, indicating that inhibiting NET formation could serve as a promising therapeutic approach for IHF-QXXY syndrome.

### Coagulation dysfunction in QXXY Syndrome

Our research uncovered significant coagulation dysfunction in IHF-QXXY syndrome patients, evidenced by reduced PGI2 levels and elevated TXA2 and TAT levels. These findings align closely with the TCM theory of “blood stasis,” indicating abnormal coagulation and microcirculatory dysfunction. IHF patients frequently experience coagulation system disorders, presenting a dual risk of both thrombosis and bleeding. As heart function declines, platelet activation becomes more pronounced [40]. This study, through multi-omics analysis, identified that platelet activation, complement and coagulation cascades, and arachidonic acid metabolism are key components in the pathological mechanisms of QXXY syndrome. Previous research has demonstrated [41] that IHF patients have shortened platelet survival times, increased mean platelet volume, and heightened platelet activation and reactivity. Additionally, increased levels of β-thromboglobulin, platelet P-selectin, cell adhesion molecules, and osteopontin further substantiate the pathological characteristics of enhanced platelet activation in IHF patients. In the proteomic analysis, we further observed the downregulation of key platelet activation-related proteins (e.g., FGA, FN1, F13A1). FGA and FN1 are crucial players in the coagulation cascade and platelet activation, and their downregulation may indicate a compensatory adjustment in coagulation function [42, 43]. However, such compensatory regulation may not fully rectify coagulation dysfunction, eventually leading to microcirculatory disturbances and tissue ischemia. The downregulation of F13A1 may impair fibrin stability, further aggravating coagulation dysfunction [44]. These findings indicate that targeting platelet activation and coagulation dysfunction could be particularly beneficial for patients with IHF-QXXY syndrome.

### Limitations and future directions

Our study provides a molecular framework for understanding QXXY syndrome within the context of modern medicine. The integration of TCM theory with multi-omics technologies offers a unique opportunity to explore the biological basis of TCM syndromes and their relevance to disease pathophysiology. The identification of key molecular pathways and biomarkers associated with QXXY syndrome not only advances our understanding of TCM but also provides a scientific basis for the development of TCM-based therapies for IHF.

While our study provides valuable insights into the molecular basis of QXXY syndrome, several limitations should be acknowledged. First, although we validated candidate biomarkers using RT-qPCR and iPRM in a subset of randomly selected samples from the discovery cohort, the absence of an independent external validation cohort limits the clinical generalizability of these findings. To enhance reliability, future studies must prioritize validation in geographically and clinically distinct cohorts, ideally through multicenter collaborations. Second, while our multi-omics network highlights potential mechanistic associations, it cannot resolve temporal dynamics or distinguish compensatory adaptations from pathogenic drivers. To address this limitation, future longitudinal studies should integrate serial multi-omics profiling (e.g., at baseline, 6-, and 12-month follow-ups) in IHF patients transitioning from early-stage QXXY syndrome to advanced heart failure. Additionally, interventional trials targeting key pathways could establish causality by examining whether modulating these pathways reverses molecular signatures and improves clinical outcomes such as LVEF or exercise capacity. Finally, the functional roles of the identified biomarkers and pathways remain to be elucidated. Experimental studies using cell and animal models are needed to validate their roles in IHF pathogenesis and to explore their therapeutic potential.

## Conclusions

Our study, through the integration of clinical samples, multi-omics research, and computational biology strategies, provides a comprehensive molecular characterization of QXXY syndrome in IHF patients. We constructed a “gene-protein-metabolite” network, comprising 6 mRNAs, 4 proteins, and 5 metabolites, which unveils key pathways and biomarkers related to energy metabolism disturbances, chronic inflammation, and coagulation dysfunction. The activation of NETs, platelet activation, complement and coagulation cascades, the HIF-1 signaling pathway, and glycolysis/gluconeogenesis, as well as the inhibition of the citrate cycle and oxidative phosphorylation, are likely hallmarks of QXXY syndrome. The syndrome is characterized by elevated levels of ACTG1, HIF-1α, P4D4, SOD2, GAPDH, and IL10, and decreased levels of F13A1, FGA, FN1, and ATP5PF. Future studies needs to validate the identified biomarkers and pathways in multicenter cohorts and longitudinal interventional trials, thereby establishing causality and therapeutic relevance.

## Supplementary Information

Additional file 1: Table S1. Data-independent-acquisition-based proteomic study. Table S2. Detailed procedures for targeted metabolomics. Table S3. Primer information. Table S4. Feature genes selected by LASSO, SVM-RFE, and RF algorithms and their intersection. Table S5. Feature proteins selected by LASSO, SVM-RFE, and RF algorithms and their intersection

## Data Availability

The data associated with this study can be obtained from the corresponding author upon reasonable request.

## Abbreviations

CI: Confidence interval
DEGs: Differentially expressed genes
DM: Differentially expressed metabolites
DEPs: Differentially expressed proteins
IHF: Ischemic heart failure
iPRM: Intelligent parallel reaction monitoring
LASSO: Least absolute shrinkage and selection operator
LVEDD: Left ventricular end-diastolic diameter
LVEDV: Left ventricular end-diastolic volume
LVEF: Left ventricular ejection fraction
MD: Mean difference
MLHFQ: Minnesota living with heart failure questionnaire
NT-proBNP: N-terminal pro-B-type natriuretic peptide
NYHA: New York Heart Association
PCA: Principal component analysis
PLS-DA: Partial least squares discriminant analysis
PPI: Protein–protein interaction network
QXXY: Qi Deficiency and Blood Stasis
RF: Random forest
ROC: Receiver operating characteristic
RT-qPCR: Real-time quantitative polymerase chain reaction
SV: Stroke volume
SVM-RFE: Support vector machine recursive feature elimination
TCM: Traditional Chinese Medicine
6 MWD: 6-Minute walking distance
