# Supplementary material for: Integrating multi-omics and machine learning strategies to explore the “gene-protein-metabolite” network in ischemic heart failure with Qi deficiency and blood stasis syndrome
Source: Chin Med. 2025 Jul 17;20:93. doi: 10.1186/s13020-025-01151-9 (PMC12269143; doi:10.1186/s13020-025-01151-9)
Supplement: Supplementary file 1 — Additional file 1: Table S1. Data-independent-acquisition-based proteomic study. Table S2. Detailed procedures for targeted metabolomics. Table S3. Primer information. Table S4. Feature genes selected by LASSO, SVM-RFE, and RF algorithms and their intersection. Table S5. Feature proteins selected by LASSO, SVM-RFE, and RF algorithms and their intersection [file 13020_2025_1151_MOESM1_ESM.docx]

**Supplementary Material**

**Table S1. Data-independent-acquisition-based proteomic study**

| Plasma protein extraction | Fasting venous blood samples were collected from participants in the morning and aliquoted into 2 ml anticoagulant tubes containing EDTA for centrifugation. The supernatant was centrifuged at 4°C at 3000 ×g for 15 minutes, collected into Eppendorf tubes, labeled, and stored at -80°C. Protein extraction and identification were conducted by Beijing Novogene Co., Ltd., using the Proteominer low-abundance protein enrichment kit (Bio-Rad, USA) to remove high-abundance proteins. Protein concentrations were measured with the Bradford Protein Assay Kit (Beyotime, China). After peptide desalting through enzymatic digestion, the samples were analyzed using LC-MS/MS. |
| --- | --- |
| LC-MS/MS Analysis-DIA mode | Prepare mobile phase A (100% water, 0.1% formic acid) and B (80% acetonitrile, 0.1% formic acid). The lyophilized powder was dissolved using 10µLA solution, centrifuged at 14,000g for 20min at 4°C, and 200 ng of the supernatant sample was injected into the sample for liquid-quality detection. The Vanquish Neo upgraded UHPLC system was used with a C18 pre-column of 174500 (5 mm×300 μm,5 μm, Thermo Fisher，USA) heated at 50°C in a column oven, and a C18 analytical column of ES906 (PepMap TM Neo UHPLC 150µm x 15 cm, 2 μm, Thermo Fisher，USA). The Orbitrap Astral masss pectrometer was used (Thermo Fisher，USA), an ESI ion source was used, the ion spray voltage was set to 1.9 kV, the ion transfer tube temperature was set to 290°C, and the mass spectrum was in a data-dependent acquisition mode, with a full first-stage mass spectrometry scanning range of m/z 380-980. The primary MS resolution was set to 240000 (200m/z), AGC was set to 500%, the parent ion window size was set to 2-Th, the number of DIA windows was 300, the NCE was set to 25%, the secondary m/z acquisition range was from 150 to 2000, the sub-ion resolution Astral was set to 80000, and the maximal injection time was 3ms. Into mass spectrometry detection raw data (.raw). |
| Trusted protein analysis | The raw files were searched and analyzed using the DIA-NN library search software, according to the homo_sapiens_uniprot_2023_10_18_Swissprot.fasta（20427 sequences） database. The library search parameters were set as follows: a mass tolerance of 10 ppm for precursor ions and 0.02 Da for fragment ions. Cysteine was modified by alkylation, methionine was oxidatively modified, and N-terminal modifications included acetylation, loss of methionine, and loss of methionine + acetylation. One missed cleavage site was allowed at most. To improve the quality of the analytical results, the DIA-NN software further filtered the search results by retaining only credible PSMs with a confidence level of 99% or higher. Only credible spectral peptides and proteins were retained, and FDR validation was performed to remove peptides and proteins with an FDR greater than 1%. |

**Table S2.** **Detailed procedures for targeted metabolomics**

| Sample preparation | Fasting venous blood samples were collected from participants in the morning and aliquoted into 2 ml anticoagulant tubes containing EDTA for centrifugation. The supernatant was centrifuged at 4°C at 3000 ×g for 10 minutes, collected into Eppendorf tubes, labeled, and stored at -80°C. Target metabolite extraction was performed. A 100 μL sample was combined with 300 μL of methanol-water solution, vortexed, and left on ice for 15 minutes. The mixture was centrifuged at 12000 rpm at 4°C for 15 minutes. A 50 μL aliquot of the supernatant was mixed with 150 μL of derivatization reagent and incubated at 40°C for 40 minutes. Subsequently, 90 μL of the supernatant was combined with 10 μL of internal standard solution, vortexed, and subjected to LC-MS analysis. Accurate quantitative analysis was conducted for 478 metabolites, including 92 amino acids, 62 aromatic compounds, 43 organic acids, 38 bile acids, 19 fatty acids, 26 carbohydrates, 24 indoles, 23 nucleosides, nucleotides, and analogs, 14 phenylpropanoids, 9 pyridines, and 128 other compounds. The metabolites were sourced from Zhenzhun Biotechnology Co., Ltd. (Shanghai), Aladdin Biochemical Technology Co., Ltd. (Shanghai), and Sigma-Aldrich (USA). |
| --- | --- |
| Standard curve establishment | Accurately weigh the standard substances of each metabolite, prepare a mixed stock solution, and dilute it with methanol to obtain a series of working solutions at different concentrations. Prepare an internal standard solution at a defined concentration, mix well, and obtain the final internal standard solution. The stock and working solutions of the linear standards, internal standards, and quality controls were stored at -20°C. Perform LC-MS analysis on the concentration series of standard solutions. The ratio of the standard concentration to the internal standard concentration is plotted on the x-axis, and the ratio of the peak area of the standard to that of the internal standard is plotted on the y-axis to evaluate the linearity of the standard solutions. |
| LC-MS/MS Analysis | UHPLC-MS/MS system (ExionLC™ AD UHPLC-QTRAP 6500+, AB SCIEX Corp., Boston, MA, USA) was used to quantitate metabolite. Separation was performed on a Waters HSS T3 column (2.1×150mm) which was maintained at 40°C. The mobile phase, consisting of 0.1% formic acid in water (solvent A) and acetonitrile/isopropanol (1:1) (solvent B), was delivered at a flow rate of 0.30 mL/min. The solvent gradient was set as follows: 5% B, 1 min; 5-40% B, 7 min; 40-95% B, 25 min; 95-5% B, 27.1 min; 30 min; 5% B. The mass spectrometer was operated in positive/negative multiple reaction mode (MRM) mode. Parameters were as follows: IonSpray Voltage (4500V/-4500V), Sheath Gas (35psi), Ion Source Temp (550°C), Auxiliary Gas (50psi), Collision Gas (55psi). |
| Data processing and analysis | After transforming the data using the metabolomics data processing software metaX, principal component analysis (PCA) and partial least squares discriminant analysis (PLS-DA) were conducted,to derive the variable importance in projection (VIP) for each metabolite.The t-test was applied to calculate the statistical significance of each metabolite between the two groups, and the fold change (FC) values for the metabolites were determined.Upregulated metabolites with an FC > 1.2, downregulated metabolites with an FC < 0.833, and VIP > 1 were used as the criteria to select DMs. |

**Table S3. Primer information**

| Gene |  | Primer | Gene ID | Amplicon Size(bp) |
| --- | --- | --- | --- | --- |
| ACTG1 | Forward | CCGAGCCGTGTTTCCTTCC | 71 | 142 |
|  | Reverse | GCCATGCTCAATGGGGTACT |  |  |
| SOD2 | Forward | TTTCAATAAGGAACGGGGACAC | 6648 | 109 |
|  | Reverse | GTGCTCCCACACATCAATCC |  |  |
| GAPDH | Forward | CCATGGGTGGAATCATATTGGA | 2597 | 138 |
|  | Reverse | TCAACGGATTTGGTCGTATTGG |  |  |
| IL-10 | Forward | GACTTTAAGGGTTACCTGGGTTG | 3586 | 112 |
|  | Reverse | TCACATGCGCCTTGATGTCTG |  |  |
| HIF-1α | Forward | GAACGTCGAAAAGAAAAGTCTCG | 3091 | 124 |
|  | Reverse | CCTTATCAAGATGCGAACTCACA |  |  |
| P4D4 | Forward | CAGGGGACATTGATCCGTGTG | 23569 | 130 |
|  | Reverse | GGGAGGCGTTGATGCTGAA |  |  |

**Table S4. Feature genes selected by LASSO, SVM-RFE, and RF algorithms and their intersection**

| Algorithm | Feature Genes Selected |
| --- | --- |
| LASSO | IGHV3-15, IGKV2D-29, IL10, CYP3A4, APOA1, PROM1, ACTG1, PFN1, PPIA, SOD2, HIF-1α, PAD4, GAPDH |
| SVM-RFE | APOA1, HIF-1α, IL10, PAD4, GAPDH, ACTG1, SOD2, CYP3A4 |
| Random forest | IGHV3-15, GAPDH, HIF-1α, IGKV2D-29, IL10, PAD4, ACTG1, SOD2 |
| Intersection Genes | HIF-1α, IL10, PAD4, ACTG1, SOD2, GAPDH |

**Table S5. Feature proteins selected by LASSO, SVM-RFE, and RF algorithms and their intersection**

| Algorithm | Feature Proteins Selected |
| --- | --- |
| LASSO | F13A1, FGA, FN1, VWF, ATP5PF |
| SVM-RFE | FGA, FGG, ATP5PF, ATP5F1D, VWF, F13A1, NDUFS5, NDUFA8, ATP5PD, CYCS, FN1, COX6B1, ATP5F1B |
| Random forest | F13A1, FN1, FGA, FGB, FGG, ATP5PF, NDUFA8 |
| Intersection Genes | F13A1, FGA, FN1, ATP5PF |
